# Protecting the Heart Without Compromising the Night— Does Cardiology Cost Sleep?

**DOI:** 10.31662/jmaj.2026-0295

**Published:** 2026-08-14

**Authors:** Atsushi Mizuno

**Affiliations:** 1 Department of Cardiovascular Medicine, St. Luke’s International Hospital, Tokyo, Japan

**Keywords:** spironolactone, esaxerenone, mineralocorticoid receptor antagonists, drug-induced insomnia, blood-brain barrier, heart failure with preserved ejection fraction (HFpEF)

While sleep has recently emerged alongside diet and exercise as an indispensable pillar of health—particularly within the realm of cardiovascular disease prevention—it remains severely underrecognized as a formal pathological entity ^(1)^. This systemic oversight is reflected in the Global Burden of Disease framework, which consistently omits sleep disorders from its core list of major diseases and risk factors. Crucially, the phenomenon of sleep impairment secondary to medical and pharmacological interventions remains poorly recognized, and the current literature suffers from a distinct paucity of comprehensive reviews examining treatment-induced or drug-induced insomnia.

Spironolactone (SPL), a first-generation mineralocorticoid receptor antagonist developed in the 1960s, features a steroidal structure that prompts off-target, non-specific binding to androgen and progesterone receptors. Given the crucial role of sex hormones in sleep regulation, this off-target activity poses a clear theoretical risk for drug-induced insomnia; however, this pathological link has long remained virtually unexamined in clinical research. Addressing this gap, Nakayama et al. conducted a retrospective cohort study using a nationwide database of 5,091 hypertensive individuals to compare the insomnia risk between SPL and esaxerenone (ESAX), a highly selective non-steroidal mineralocorticoid receptor antagonist (MRA) ^(2)^. Over a median follow-up of 512 days, 356 incident insomnia events were documented. Propensity-score-weighted analysis revealed that the initiation of SPL was associated with a significantly elevated risk of developing insomnia compared to ESAX (hazard ratio [HR], 1.66; 95% confidence interval, 1.32-2.10), a trend that remained consistent in both men (HR, 1.79) and women (HR, 1.59).

Even when accounting for nocturia—a common class effect of MRAs, which are alternatively classified as potassium-sparing diuretics and inherently alter fluid dynamics—the stark divergence in insomnia risk underscores a critical, drug-specific phenomenon. The clinical insight that incident insomnia occurs even in a population with no prior sleep disorders highlights a vital real-world reality. This suggests that the observed differences between these agents extend far beyond simple volume depletion or class-wide diuretic effects, pointing instead to a distinct central mechanism.

The molecular underpinnings of this drug effect lie in the interplay between blood–brain barrier (BBB) permeability and receptor selectivity. SPL, owing to its lipophilic steroidal structure, readily crosses the BBB and enters the central nervous system (CNS). Once inside the brain, it exhibits off-target binding to progesterone receptors, disrupting the neurosteroid pathways that normally enhance gamma-aminobutyric acid-mediated inhibitory drive. By blunting this vital central sleep drive, SPL promotes hyperarousal and induces insomnia. In stark contrast, ESAX features a novel non-steroidal structure that inherently limits its BBB permeability, minimizing CNS entry. Furthermore, its high selectivity for the mineralocorticoid receptor ensures zero off-target interaction with sex hormone receptors, delivering robust cardiorenal protection without compromising sleep. Although the retrospective nature of the current study cannot definitively establish direct causality, these robust pharmacological mechanisms provide compelling theoretical support, warranting further prospective verification in future research.

This mechanistic divergence holds profound clinical implications, especially as non-steroidal MRAs rapidly gain prominence in the management of heart failure with preserved ejection fraction (HFpEF). Unlike SPL, which yielded mixed results in the historic TOPCAT trial, newer non-steroidal MRAs—most notably finerenone—have recently demonstrated definitive efficacy in improving clinical outcomes for patients with HFpEF ^(3)^. This clinical breakthrough has shifted the spotlight toward the unique therapeutic value of the non-steroidal class. While contemporary randomized controlled trials (RCTs) are heavily optimized for these hard endpoints, unmeasured outcomes such as sleep quality and iatrogenic insomnia have long been left within a “black box.” Crucially, sleep impairment is not merely a quality-of-life issue; it is a powerful prognostic determinant. Chronic insomnia triggers sympathetic hyperactivity and a non-dipping blood pressure profile, paradoxically exacerbating the very cardiovascular risks clinicians intend to mitigate.

Shedding light on this black box using real-world data is both scientifically compelling and pathophysiologically plausible. While traditional RCTs remain the gold standard for evaluating efficacy, this study exposes a broader, systemic challenge in modern medicine: how to properly integrate sleep—an indispensable pillar of holistic health—into the evaluation of cardiovascular therapies. Currently, clear frameworks for assessing and managing sleep health within cardiovascular clinical trials remain undefined. Recognizing SPL-induced insomnia justifies immediate, pragmatic strategies like intraclass switching to ESAX. Moving forward, however, we must transcend the rigid confines of hard endpoints and establish clear methodologies to measure sleep quality, ensuring that our evidence-based efforts to protect the heart do not inadvertently silence the quiet of the night.

## Article Information

### Conflicts of Interest

None

### Disclaimer

Atsushi Mizuno is one of the Editors of JMA Journal and on the journal’s Editorial Staff. He was not involved in the editorial evaluation or decision to accept this article for publication at all.
